# Glucagonoma Syndrome With Necrolytic Migratory Erythema

**DOI:** 10.7759/cureus.99747

**Published:** 2025-12-20

**Authors:** Hiebda Sofía Martínez Jiménez

**Affiliations:** 1 Internal Medicine, Centro Médico Nacional Siglo XXI, Mexico City, MEX

**Keywords:** glucagonoma, glucagonoma syndrome, interests in internal medicine, internal diseases, necrolytic migratory erythema

## Abstract

Glucagonoma is a rare pancreatic alpha-cell tumor that leads to glucagonoma syndrome, typically characterized by necrolytic migratory erythema (NME), diabetes, weight loss, and anemia. We present the case of a 47-year-old man with a one-year history of diabetes and a relapsing pustular and scaly dermatosis. Examination revealed widespread annular erythematous plaques. Laboratory tests showed anemia, hypoalbuminemia, and hyperglycemia. Skin biopsy demonstrated epidermal necrolysis, and abdominal imaging identified a 3.1 cm tumor in the pancreatic tail. Surgical resection confirmed a well-differentiated neuroendocrine tumor (Ki67: 1%), and skin lesions resolved postoperatively. This case highlights an atypical presentation of NME and the importance of early recognition to reduce diagnostic delay in glucagonoma syndrome.

## Introduction

Glucagonoma is an alpha‐cell‐secreting tumor of the pancreas that secretes glucagon and causes glucagonoma syndrome. Common features of glucagonoma syndrome include necrolytic migratory erythema (NME), diabetes, weight loss, and anemia, of which NME is the first clinical finding in 70% of individuals with glucagonoma syndrome [1]. NME secondary to glucagonoma is an exceptionally rare paraneoplastic dermatosis that frequently presents a diagnostic challenge [2]. Clinically, this cutaneous eruption typically follows a relapsing-remitting course. It is referred to as NME owing to its migratory pattern, erythematous presentation, and histopathological features that include necrosis of the upper layers of the spinous epidermis [3,4]. We present the case of a man with an atypical dermatological presentation of glucagonoma syndrome, which included NME.

## Case presentation

A 47-year-old man developed erythematous scaly plaques and pustules with a relapsing and recurring course in 2019. He had been diagnosed with diabetes one year previously. He was referred to a tertiary care center for comprehensive evaluation.

**Figure 1:**
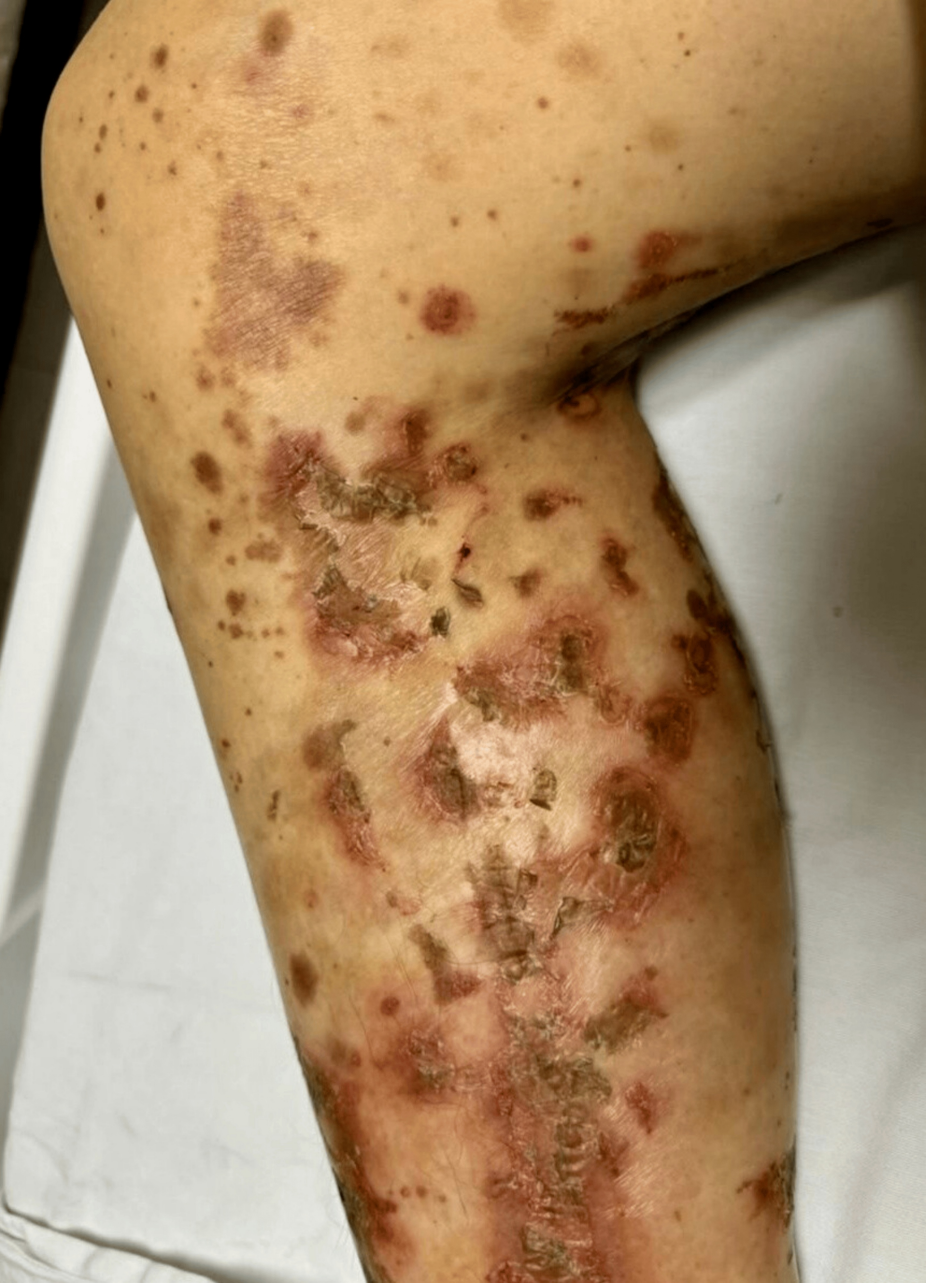
Necrolytic migratory erythema involving the extremities

**Figure 2:**
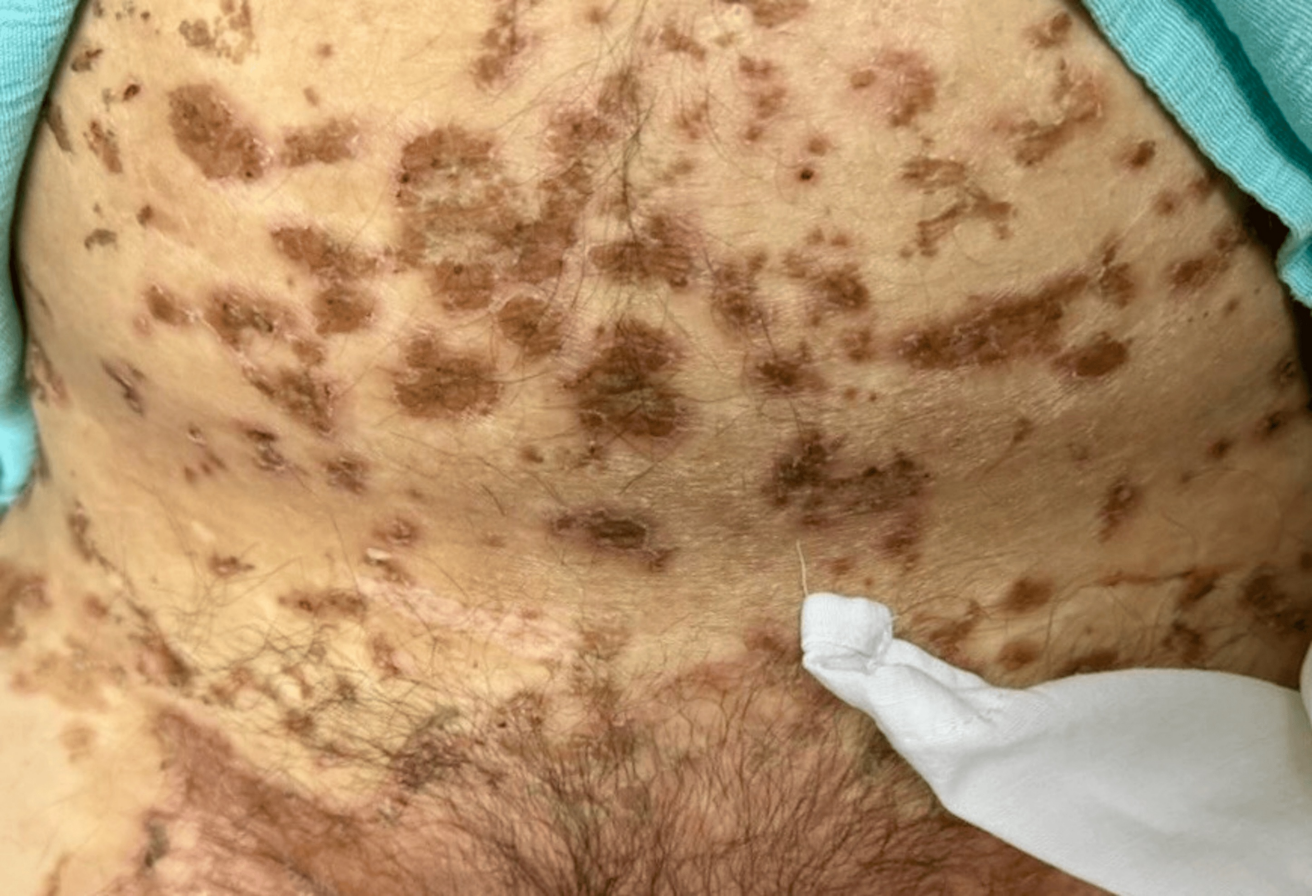
Necrolytic migratory erythema involving the trunk

Physical examination revealed extensive dermatosis involving the thoracic and pelvic limbs, bilateral inguinal region, anterior and posterior thorax, abdomen, lumbar, genital, and intergluteal regions, characterized by erythematous-violaceous plaques in an annular configuration with thick scales and well-defined borders (Figures 1, 2). Laboratory tests showed hypoalbuminemia, elevated fasting blood glucose, and anemia (Table 1).

**Table 1:** Laboratory parameters and reference ranges

Parameters	Result	Reference Value
Glucose	136 mg/dL	70-105 mg/dl
Urea	52.1 mg/dL	16.6-48.5 mg/dL
Blood Urea Nitrogen (BUN)	24.3 mg/dl	8.9-20.6 mg/dL
Creatinine	0.58 mg/dL	0.72-1.25 mg/dL
Albumin	2.2 g/dL	3.5-5.0 g/dL
White Blood Cells (WBC)	8.94 10x3/uL	4.60-10.20
Hemoglobin	10.5 g/dL	13-18 g/dL
Mean Corpuscular Volume (MCV)	106.5 fL	90-97 fL
Mean Corpuscular Hemoglobin (MCH)	34.6 pg	27-31 pg
Platelets	207 10x3 /uL	150-450
Neutrophils	8.26 10x3/uL	1.5-7

**Figure 3:**
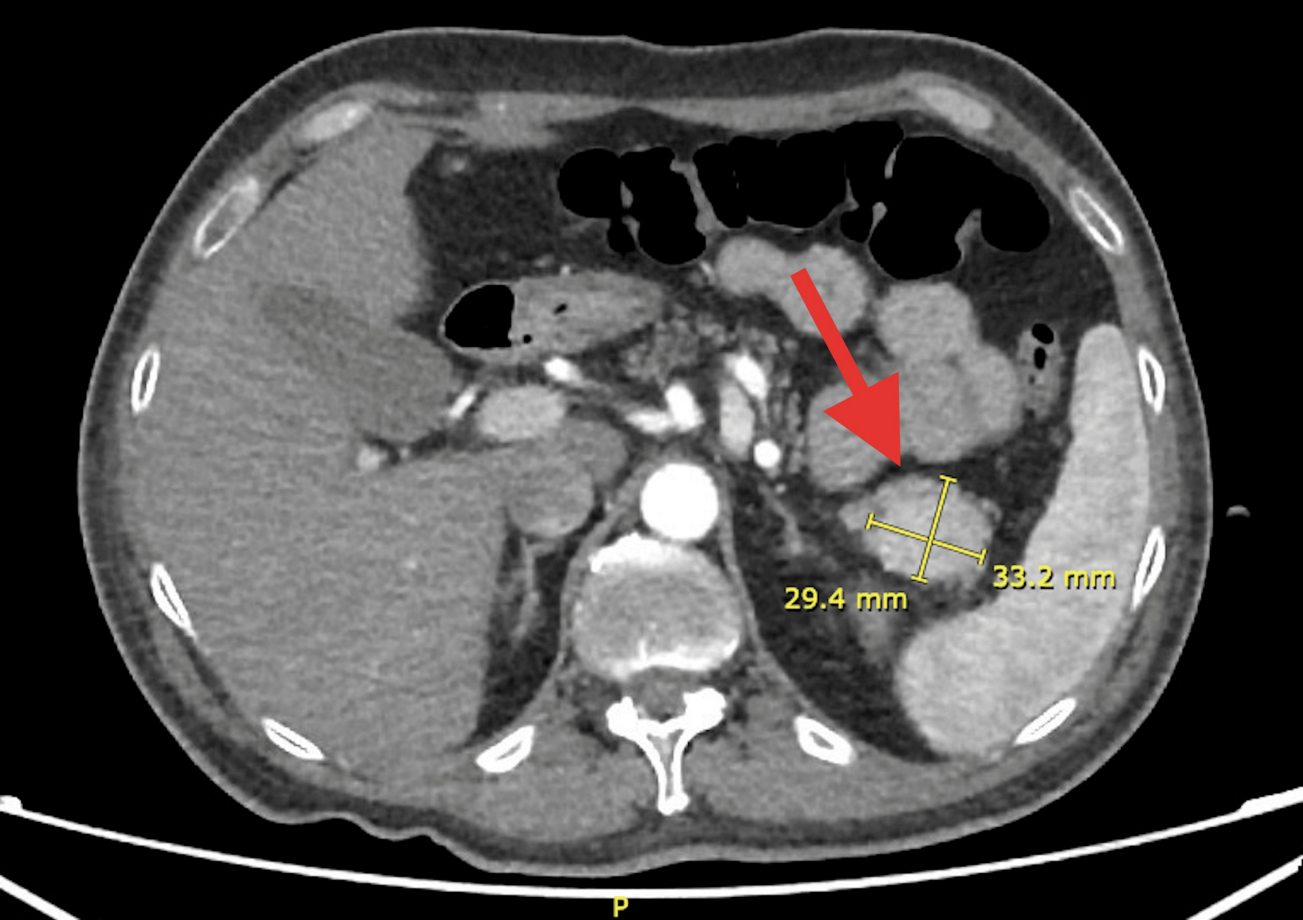
Glucagonoma located in the tail of the pancreas on abdominal CT Abdominal contrast-enhanced CT scan revealing a well-defined, heterogeneous mass located in the tail of the pancreas, measuring 29.4 mm × 33.2 mm, consistent with a glucagonoma (red arrow).

**Figure 4:**
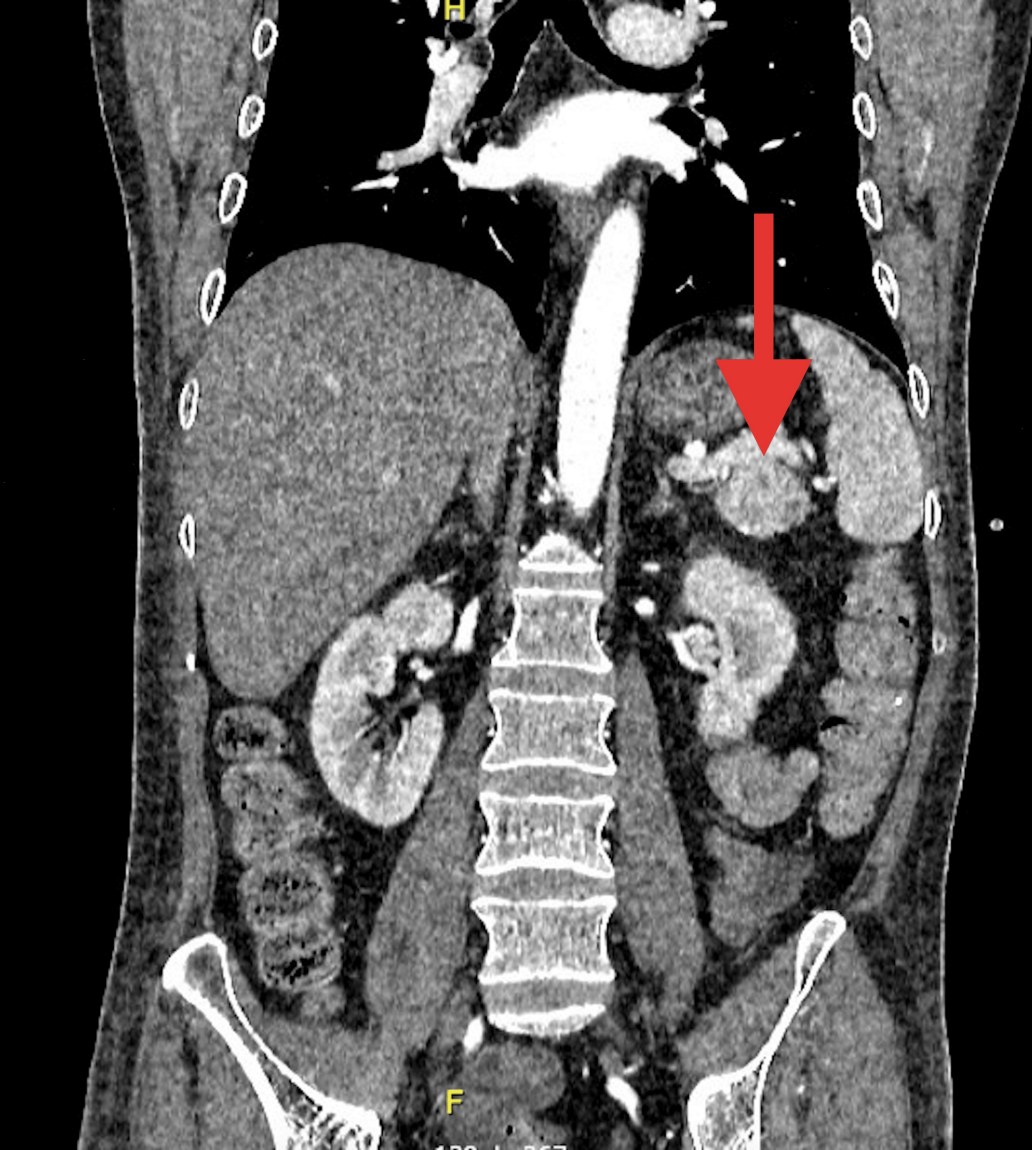
Coronal computed tomography scan highlighting a pancreatic glucagonoma This coronal CT scan depicts a well-defined mass in the tail of the pancreas, indicated by the red arrow, consistent with a glucagonoma.

Based on these findings, the patient was presumptively diagnosed with NME. Abdominal computed tomography showed a tumor in the tail of the pancreas measuring 29.4 mm x 33.2 mm with heterogeneous enhancement (Figures 3, 4). 

A skin biopsy and abdominal computed tomography were performed to confirm the diagnosis. The skin biopsy showed epidermal necrolysis, vacuolated keratinocytes in the upper epidermis, and perivascular lymphocytic and histiocytic infiltrates in the dermis. Microscopic images are not available.

A distal pancreatectomy was performed to remove the tumor. Preoperative imaging studies revealed no evidence of distant metastases. Histopathology confirmed a well-differentiated grade I neuroendocrine tumor with a Ki67 proliferation index of 1%. During hospitalization, diabetes was managed with 10 units of insulin glargine daily, achieving adequate glycemic control. After the removal of the tumor, the skin lesions resolved within weeks, leaving post-inflammatory hyperpigmentation.

## Discussion

Glucagonoma syndrome is a rare disease with an estimated incidence of one in 20 million; given the infrequency of glucagonoma syndrome, it is often overlooked by clinicians, who may mistake its cutaneous manifestations for more prevalent conditions like intertrigo, contact dermatitis, or psoriasis [2]. Most glucagonomas are sporadic, but approximately 3% are associated with genetic syndromes; hereditary glucagonomas are typically non-functional [5]. In around 87% of cases, the glucagon-producing tumor is found in the body or tail of the pancreas, while the rest are situated predominantly in the head of the pancreas [6]. NME is the most characteristic cutaneous manifestation of glucagonoma syndrome [7]. NME has also rarely been reported in the absence of a pancreatic tumor; this presentation, referred to as “pseudo-glucagonoma” syndrome, has been most frequently linked to celiac disease and malabsorption, followed by associations with cirrhosis and non-islet cell tumors. Other reported conditions include pancreatitis, hepatitis, and inflammatory bowel disease [8]. 

NME commonly affects the perineum and other intertriginous sites. The trunk, legs, perioral skin, and sites of minor trauma can also be involved [9]. The typical lesions of NME initially appear as pruritic, irregular erythematous lesions with subsequent necrosis and central crusting, leading to bullae and healing with hyperpigmentation. Pustules are an atypical manifestation [10].

When evaluating laboratory findings, fasting glucagon levels are considered highly specific for diagnosing glucagonoma, with values above 500 pg/mL typically confirming the diagnosis (normal range: 70-160 pg/mL). In addition, several biochemical markers are useful for both diagnosis and follow-up. Among the non-specific markers, chromogranin A is associated with well-differentiated neuroendocrine tumors, neuron-specific enolase serves as a marker for poorly differentiated tumors, and pancreatic polypeptide is often elevated in non-functioning pancreatic neoplasms [11].

The gold standard for the diagnosis of glucagonoma syndrome is selective visceral angiography, which enables precise localization of the tumor by evaluating the celiac axis, superior mesenteric artery, and branches supplying the pancreas [6]. The histological findings of NME can be nonspecific, with the most common finding being superficial epidermal necrolysis with vacuolated keratinocytes [5].

The time from symptom onset to diagnosis is generally two to four years, and 49.2% of patients have metastases at the time of diagnosis [12]. The definitive treatment for glucagonoma and NME is surgical removal of the tumor. Cutaneous symptoms typically disappear within a few days following complete surgical excision of the tumor. Somatostatin, a regulatory peptide secreted by gastrointestinal paracrine cells, suppresses the secretion of various hormones, including glucagon, gastrin, and insulin. Long-acting somatostatin analogs, such as octreotide-long-acting release (LAR), are effective in managing clinical manifestations and are generally well tolerated by patients [3]. Treatment with somatostatin analogues may result in clinical resolution, particularly in tumors with somatostatin receptor expression [6], and may be used as an alternative to surgery in selected cases, especially when the tumor is unresectable or the patient is not a surgical candidate.

## Conclusions

Due to its rarity and the nonspecific nature of its clinical manifestations, the diagnosis of glucagonoma syndrome is frequently delayed, often leading to misdiagnosis of its hallmark skin manifestations as more common dermatologic conditions such as psoriasis, intertrigo, or contact dermatitis. This diagnostic challenge underscores the importance of heightened clinical suspicion, especially in patients presenting with persistent, atypical, or relapsing cutaneous eruptions combined with systemic symptoms like diabetes and weight loss. Early recognition of NME as a paraneoplastic sign can prompt timely imaging and biochemical investigations, facilitating earlier diagnosis and treatment.
